# Supplementary material for: Assessing the value of deep neural networks for postoperative complication prediction in pancreaticoduodenectomy patients
Source: PLoS One. 2024 Dec 30;19(12):e0316402. doi: 10.1371/journal.pone.0316402 (PMC11684602; doi:10.1371/journal.pone.0316402)
Supplement: S1 Data — (DOCX) [file pone.0316402.s001.docx]

# Supplementary data

|  | **General dataset**  **(n=5,874,941)** | **PD dataset (n=17,037)** | **Test dataset (n=2,000)** |
| --- | --- | --- | --- |
| **Continues Variables** |  |  |  |
| Height | 66.00 (1.96%) | 66.00  (0.46%) | 66.00  (0.35%) |
| Weight | 180.00  (1.11%) | 167.00  (0.25%) | 168.00  (0.05%) |
| Age | 58.00  (0.00%) | 66.00  (0.00%) | 66.00  (0.00%) |
| Operation Time | 85.00  (0.01%) | 354.00  (0.0%) | 360.00  (1.2%) |
| Days from Hospital Admission to Operation | 0.00  (0.00%) | 0.00  (0.00%) | 0.00  (0.00%) |
| Work Relative Value Units (wRVUs) | 15.37  (0.00%) | 52.84  (0.00%) | 52.84  (0.00%) |
| Serum Sodium | 139.00  (19.42%) | 139.00  (2.07%) | 139.00  (2.55%) |
| Serum Blood Urea Nitrogen | 15.00  (22.74%) | 14.00  (5.48%) | 14.00  (5.45%) |
| Serum Creatinine | 0.85  (18.55%) | 0.80  (1.72%) | 0.80  (2.35%) |
| Serum Albumin | 4.00  (49.04%) | 3.80  (8.22%) | 3.80  (8.25%) |
| Serum Bilirubin | 0.50  (49.49%) | 0.70  (7.77%) | 0.70  (9.05%) |
| Serum Glutamic-Oxaloacetic Transaminase | 21.00  (49.72%) | 29.00  (7.01%) | 29.00  (7.85%) |
| Serum Alkaline Phosphatase | 76.00  (49.25%) | 119.00  (7.21%) | 120.00  (7.95%) |
| Serum White Blood Cell Count | 7.33  (16.49%) | 6.93  (1.79%) | 7.10  (2.20%) |
| Serum Hematocrit | 40.00  (15.19%) | 38.00  (1.64%) | 37.90  (2.15%) |
| Serum Platelet Count | 240.00  (16.54%) | 245.00  (1.82%) | 247  (2.35%) |
| Thromboplastin time | 29.10  (68.35%) | 29.40  (36.76%) | 29.60  (38.70%) |
| INR | 1  (59.09%) | 1  (17.40%) | 1  (17.75%) |
| Prothrombin Time | 12.30  (98.49%) | 12.50  (96.48%) | 13.35  (96.90%) |
| **Categorical variables** |  |  |  |
| Sex  Male  Female | 3,331,363 (56.96%)  2,517,490 (43.04%) | 7,963 (46.74%)  9,074 (53.26%) | 918 (45.90%)  1,082 (54.10%) |
| Functional Status  Independent  Partially dependent  Totally dependent | 5,653,044 (96.65%)  129,552 (2.21%)  27,193 (0.46%) | 16,844 (98.87%)  145 (0.85%)  12 (0.07%) | 970 (98.50%)  18 (0.90%)  1 (0.05%) |
| ASA  1  2  3  4  5 | 513,395 (8.78%)  2,621,823 (44.83%)  2,342,428 (40.05%)  345,329 (5.90%)  10,514 (0.18%) | 78 (0.46%)  3,748 (22.00%)  11,952 (70.15%)  1,236 (7.25%)  1 (0.01%) | 15 (0.75%)  410 (20.50%)  1,428 (71.40%)  145 (7.25%)  1 (0.05%) |
| Steroid use  No  Yes | 5,636,210 (96.36%)  212,644 (3.64%) | 16,563 (97.22%)  474 (2.78%) | 1,932 (96.60%)  68 (3.40%) |
| Ascites  No  Yes | 5,828,908 (99.66%)  212,644 (3.64%) | 16,980 (97.22%)  474 (2.78%) | 1,994 (99.70%)  68 (3.40%) |
| System Sepsis  None  Systemic Inflammatory response syndrome  Sepsis Septic Shock | 5,530,373 (94.55%)  174,477 (2.98%)  120,726 (2.06%)  23,271 (0.40%) | 168,07 (98.65%)  149 (0.87%)  75(0.44%)  6 (0.04%) | 1,969 (98.45%)  27 (1.35%)  4 (0.20%)  0 (0.00%) |
| Ventilator Dependent  No  Yes | 5,829,896 (99.68%)  18,958 (9.32%) | 17,029 (99.95%)  8 (0.05%) | 1,998 (99.90%)  2 (0.10%) |
| Disseminated cancer  No  Yes | 5,716,594 (97.74%)  132,261 (2.26%) | 16,200 (95.09%)  837 (4.91%) | 1,910 (95.50%)  90 (4.50%) |
| Diabetes  No  Non-insulin dependent  Insulin dependent | 4,946,250 (84.57%)  566,979 (9.69%)  335,623 (5.74%) | 12,575 (73.81%)  2,249 (13.20%)  2,213 (12.99%) | 1,464 (73.20%)  276 (13.80%)  260 (13.00%) |
| Hypertension  No  Yes | 3,226,196 (55.16%)  2,622,659 (44.84%) | 7,806 (45.82%)  9,231 (54.81%) | 935 (46.75%)  1,065 (53.25%) |
| Congestive Heart Failure  No  Yes | 5,798,640 (99.14%)  50,215 (0.86%) | 16,969 (99.60%)  68 (0.40%) | 1,990 (99.50%)  10 (0.50%) |
| Dyspnea  No  Moderate Exertion  At Rest | 5,521,674 (94.41%)  301,183 (5.15%)  25,994 (0.44%) | 16,120 (94.62%)  886 (5.20%)  31 (0.18%) | 1,881 (94.05%)  118 (5.90%)  1 (0.05%) |
| Current Smoker  No  Yes | 4,823,461 (82.47%)  1,025,393 (17.53%) | 13,879 (81.46%)  3,158(18.54%) | 1,634 (81.70%)  366 (18.30%) |
| History of COPD  No  Yes | 5,589,799 (95.57%)  259,056(4.43%) | 16,338 (95.90%)  699 (4.10%) | 1,926 (96.30%)  74 (3.70%) |
| Dialysis  No  Yes | 5,770,953 (98.67%)  77,902 (1.33%) | 16,974 (99.63%)  63 (0.37%) | 1,995 (99.75%)  5 (0.25%) |
| Acute Renal Failure  No  Yes | 5,82,8481 (99.65%)  20,373(0.35%) | 17,023 (99.92%)  14 (0.08%) | 1,999 (99.95%)  1 (0.05%) |
| Operation Year  2012  2013  2014  2015  2016  2017  2018 | 540,071 (9.23%)  647,188 (11.07%)  746,156 (12.76%)  880,757 (15.06%)  995,438 (17.02%)  1,023,700 (17.50%)  1,015,547 (17.36%) | 1,736 (10.19%)  2,101 (12.33%)  2,240 (13.15%)  2,551 (14.97%)  2,722 (15.98%)  2,819 (16.55%)  2,868 (16.83%) | 204 (10.20%)  220 (11.00%)  266 (13.30%)  307 (15.35%)  305 (15.25%)  343 (17.15%)  355 (17.75%) |
| Race  White  African American  Asian  American Indian or Alaska Native  Native Hawaiian or Pacific Islander | 4,185,428 (71.56%)  582,001 (9.95%)  165,649 (2.83%)  33,389 (0.57%)  22,730 (0.39%) | 12,926 (75.87%)  1,480 (8.69%)  663 (3.89%)  50 (0.29%)  36 (0.21%) | 1,526 (76.30%)  181 (9.05%)  59 (2.95%)  5 (0.25%)  7 (0.35%) |
| Hispanic Ethnicity  No  Yes | 4,559,006 (77.95%)  448,086 (7.66%) | 14,593 (85.65%)  906 (5.32%) | 1,714 (85.70%  101 (5.05%) |
| Principal Anesthesia Technique  General  Spinal  MAC/IV Sedation  Regional  Local Epidural  Other | 5,214,299 (89.15%)  294,235 (5.03%)  273,559 (4.68%)  38,310 (0.65%)  13,839 (0.24%)  8,182 (0.14%)  4,358 (0.07%) | 16,946 (99.47%)  9 (0.05%)  9 (0.05%)  5 (0.03%)  1 (0.01%)  41 (0.24%)  21 (0.12%) | 1,992 (99.60%)  0 (0.00%)  2 (0.10%)  1 (0.05%)  0 (0.00%)  3 (0.15%)  2 (0.10%) |
| Wound Classification  Clean  Clean/Contaminated Contaminated  Dirty/infected | 3,297,730 (53.38%)  1,899,222 (32.47%)  359,186 (6.14%)  292,715 (5.00%) | 463 (12.97%)  13,887 (81.51%)  2,209(12.97%)  478 (2.81%) | 52 (2.60%)  1,619 (80.95%)  270 (13.50%)  59 (2.95%) |
| Wound infection/open  No  Yes | 5,678,499 (97.09%)  170,356 (2.91%) | 169,53 (99.51%)  84 (0.49%) | 1,989 (99.45%)  11 (0.55%) |
| Elective Surgery  No  Yes | 1,183,627 (20.24%)  4,658,162 (97.09%) | 1,624 (9.53%)  15,404 (90.41%) | 176 (8.80%)  1,823 (91.15%) |
| Bleeding disorder  No  Yes | 5,605,529 (95.84%)  243,326 (4.16%) | 16,584 (97.34%)  453 (2.66%) | 1,950 (97.50%)  50 (2.50%) |
| Preoperative Weight Loss  No  Yes | 5,779,776 (98.82%)  69,078 (1.18%) | 14,454 (84.84%)  2,583 (15.16%) | 1,662 (83.10%)  338 (16.90%) |
| Preoperative blood transfusion  No  Yes | 5,795,735 (99.09%)  53,119 (0.91%) | 16,863 (98.98%)  174 (1.02%) | 1,985 (99.25%)  15 (0.75%) |
| In/Out – Patient Status  Inpatient  Outpatient | 3,419,449 (58.46%)  2,429,407 (41.54%) | 17,000 (99.78%)  37 (0.22%) | 1,995 (99.75%)  5 (0.25%) |
| Transfer Status  Not transferred  Outside Emergency Department  From acute care hospital inpatient  Nursing Home Transfer from other | 5,586,747 (95.52%)  97,228 (1.66%)  86,515 (1.48%)  54,747 (0.94%)  16,695 (0.29%) | 16,484 (96.75%)  72 (0.42%)  406 (2.38%)  38 (0.22%)  33 (0.19%) | 1,952 (97.60%)  4 (0.20%)  38 (1.90%)  3 (0.15%)  2 (0.10%) |
| Superficial SSI PATOS  No  Yes | 5,843,176 (99.90%)  5,357 (0.10%) | 16,988 (99.71%)  49 (0.29%) | 1,995 (99.75%)  5 (0.25%) |
| Deep SSI PATOS  No  Yes | 5,841,446 (99.87%)  7,387 (0.13%) | 17,013 (99.86%)  24 (0.14%) | 1,997 (99.85%)  3 (0.15%) |
| Organ/space SSI PATOS  No  Yes | 5,824353 (99.58%)  24,480 (0.42%) | 16,737 (98.24%)  300 (1.76%) | 1,959 (97.95%)  41 (2.05%) |
| Pneumonia PATOS  No  Yes | 5,835,621 (99.77%)  24,480 (0.42%) | 17,015 (99.87%)  22 (0.13%) | 1,997 (99.85%)  3 (0.15%) |
| On Ventilator > 48 hours PATOS  No  Yes | 5,837,288 (99.80%)  11,545 (0.20%) | 17,026 (99.94%)  11 (0.06%) | 1,998 (99.90%)  2 (0.10%) |
| UTI PATOS  No  Yes | 5,836,542 (99.79%)  12,291 (0.21%) | 17,011 (99.85%)  26 (0.15%) | 1,995 (99.75%)  5 (0.25%) |
| SEPSIS PATOS  No  Yes | 5,798,673 (99.14%)  50,160 (0.86%) | 16,664 (97.81%)  373 (2.19%) | 1,946 (97.30%)  54 (2.70%) |
| SEPTIC SHOCK PATOS  No  Yes | 5,826,239 (99.61%)  22,594 (0.39%) | 16,993 (99.74%)  44 (0.26%) | 1,998 (99.90%)  2 (0.10%) |

**Table 1**: Input variables for the three dataframes before the split into validation/training sets are depicted above. Continuous variables are represented by median values, with the percentage of missing data in parentheses. Categorical variables show the count in each category, with the total percentage in parentheses, with the remaining being missing data.

PATOS: Present at time of surgery

| **NRI** | **Transfer-learning model vs Direct model** | **Transfer-learning model vs Random Forest model** | |
| --- | --- | --- | --- |
| **Superficial Surgical site infection** | 0.076900 | | 0.0 |
| **Deep surgical site infection** | 0.087193 | | 0.00029 |
| **Organ/space surgical site infection** | 0.015263 | | 0.00088 |
| **Wound disruption** | 0.160845 | | 0.0 |
| **Postoperative pneumonia** | 0.078368 | | 0.0 |
| **Unplanned intubation** | 0.124450 | | -0.00029 |
| **Pulmonary embolism** | 0.108600 | | 0.00029 |
| **Ventilator dependence >48 hours** | 0.167890 | | 0.02260 |
| **Progressive renal insufficiency** | 0.130026 | | -0.92603 |
| **Acute renal failure** | 0.187262 | | -0.10507 |
| **Urinary tract infection** | 0.028177 | | 0.00059 |
| **Stroke** | 0.121808 | | -0.94687 |
| **Cardiac arrest** | 0.198122 | | 0.00264 |
| **Myocardial infarction** | 0.104784 | | -0.68271 |
| **Deep vein thrombosis** | 0.108600 | | 0.00029 |
| **Sepsis** | -0.024949 | | 0.00029 |
| **Septic shock** | 0.137364 | | 0.10566 |
| **Bleeding requiring transfusion** | 0.073378 | | 0.00558 |
| **Death** | 0.247138 | | 0.02554 |

**Table 2**: Net Reclassification Index (NRI) values the Transfer-learning model vs the direct model is based on a threshold on 0.5. The Transfer-learning model vs Random Forest is based on a threshold of 0.01.
