# Supplementary material for: Assessing the value of deep neural networks for postoperative complication prediction in pancreaticoduodenectomy patients
Source: PLoS One. 2024 Dec 30;19(12):e0316402. doi: 10.1371/journal.pone.0316402 (PMC11684602; doi:10.1371/journal.pone.0316402)
Supplement: Attachment — Submitted filename: Response to reviewers.docx [file pone.0316402.s002.docx]

Copenhagen March 19th 2024

Dear Dr. Chen

Thank you for the opportunity to resubmit our manuscript. Please find a point-by-point response to reviewers’ comments below. Also, please do not hesitate to reach out to us if further information is required.

On behalf of the authors

Martin Sillesen MD, PhD

Assoc. Prof. of Surgery

Dep. of Organ Surgery and Transplantation
Copenhagen University Hospital, Rigshospitalet
Denmark

[Martin.Sillesen@Regionh.dk](mailto:Martin.Sillesen@Regionh.dk)

Journal Requirements:

When submitting your revision, we need you to address these additional requirements.

Comment #1

1. Please ensure that your manuscript meets PLOS ONE's style requirements, including those for file naming. The PLOS ONE style templates can be found at 
https://journals.plos.org/plosone/s/file?id=wjVg/PLOSOne_formatting_sample_main_body.pdf and 
https://journals.plos.org/plosone/s/file?id=ba62/PLOSOne_formatting_sample_title_authors_affiliations.pdf

Response:
Thank you for highlighting this. The formatting issues have been addressed.

Comment #2

2.  Please provide additional details regarding participant consent. In the ethics statement in the Methods and online submission information, please ensure that you have specified what type you obtained (for instance, written or verbal, and if verbal, how it was documented and witnessed). If your study included minors, state whether you obtained consent from parents or guardians. If the need for consent was waived by the ethics committee, please include this information.

Response:
Thank you for this question. The project received a waiver of IRB approval from the Massachusetts General Hospital IRB. This is stated in the methods section. We have expanded this section further to detail that this entailed that patient consent was not required and thus not obtained.

Comment #3

3. Thank you for stating the following in the Competing Interests section: "I have read the journal's policy and the authors of this manuscript have the following competing interests: Authors AB and MS have founded Aiomic Aps, a healthtech company fielding artificial intelligence models for healthcare use. The present work is for research only and is not related to any commercial activities."

Response:

You are welcome

Comment #4

Please confirm that this does not alter your adherence to all PLOS ONE policies on sharing data and materials, by including the following statement: "This does not alter our adherence to  PLOS ONE policies on sharing data and materials.” (as detailed online in our guide for authors http://journals.plos.org/plosone/s/competing-interests).  If there are restrictions on sharing of data and/or materials, please state these. Please note that we cannot proceed with consideration of your article until this information has been declared.

Please include your updated Competing Interests statement in your cover letter; we will change the online submission form on your behalf.

Response:

Thank you, we have updated the cover letter with the required information.

Reviewers' comments:

Reviewer's Responses to Questions

**Comments to the Author**

Reviewer #1:

Comment #5

I appreciate the opportunity to examine this interesting paper. Nevertheless, there are a few concerns regarding the work.
Conduct a comprehensive review of existing literature pertaining to the utilization of machine learning and deep learning techniques in the context of mortality and morbidity, as well as any relevant studies in related fields. The utilization of Deep Neural Networks (DNN) and Random Forest (RF) can be justified based on their respective advantages and capabilities. There are numerous AI algorithms available, with the latest one being ensemble learning, which offers superior performance. Deep neural networks (DNN) may not consistently perform as effectively as other machine learning methods, but they are particularly notable for their application in image processing. This is evident when the area under the curve (AUC) has a relatively low value. A value that is considered acceptable is 0.75 or higher.
The authors should explicitly state their objective and anticipated outcome in the objective section. As I comprehend, there are multiple morbidity outcomes and death outcomes, and the authors conducted different modeling for each result. Alternatively, is it a prediction involving many classes? The authors should explicitly articulate this.

Response:
Thank you these comments. Overall, we fully agree with the reviewer that there are multiple different modeling options well suited for this prediction task. We have chosen the DNN architecture, as we have previously demonstrated superior performance using this approach on a general surgical dataset (Manuscript reference #12).
In this modeling study, we use the DNNs to do multiclass prediction, so it is essentially the same network used for multi-label prediction of several different postoperative complications. We have augmented the manuscript with a further description of this important point, as well as elaborated on the expected outcome in the introduction as requested.

Comment #6
In the data section, specify the percentage of missing values for final variables and identify the data imputation method employed.

Response :
Thank you for this question. We agree that the issue of missing data is important. For the final (output) variables, the dataset was complete and thus did not have missing data. This is because the NSQIP database does not allow for incomplete postoperative complication data entry when submitting cases. We have added this information to the methods section of the manuscript, as well as included a description of how missing data was handled for input variables. For the input data, an overview of demographic variables between datasets, as well as the percentage of missing data, has now been included in the supplementary data table 1.

Comment #7

The authors determined the final features from the 150 variables listed in the study. The ultimate result must also be explicitly expressed. It is necessary to provide a clear explanation of how the dataset is allocated to the four models. How did the authors address the issue of data imbalance, which can explain the low AUC value? There is a noticeable imbalance in the data when merging patients with PD with people without PD.

Response:

Thank you for this insightful question. We agree that a full overview of the input features is important for assessing the data on which the model performs its predictions. We have added a table detailing the input features as supplementary data.
The reviewer is indeed correct in stating that the class imbalance in the data may well impact on the AUC performance score. This problem often occurs in postoperative complication data, as the outcomes are infrequent. We have, however, sought to address this through the introduction of positive weights applied to the loss function of the DNN. This approach has been further detailed in the methods section

Comment #8
Summary statistics of each dataset are compared against the outcome using chi square analysis, yielding the chi square value for categorical and corresponding p-value continuous variable.

Response:

In this study, we have not calculated summary statistics of the different datasets for several reasons. First, the objective is not to determine whether significant differences existed between input and output variables. This is no doubt the case, as a general surgical cohort will for example have a different spectrum and incidence of postoperative complications as would a specific pancreatic cohort. We thus feel that the objective of the study is not to compare cohorts and identify differences in these, but rather to investigate how optimal training of a PD specific model can be achieved.
Even if it was chosen that summary statistics were required, the number of input and output variables would necessitate correction for multiple testing due to the number of variables included. As such, there would be a clear risk of a Type-II error, which again would diminish the usefulness of such a comparison. While this can in certain cases of cause be acceptable, we respectfully feel that a comparison of cohorts is not within the objective of this study. Rather this is a model comparison study.

Comment #9
Did the authors undertake parameter optimization on the RF model to enhance its performance? Support Vector Machines (SVM) are a suitable option for conducting benchmarking.

Response:
Thank you for this comment. We agree that the SVM approach could be useful a study such as this. As suggested, we have benchmarked the RF models with an SVM approach with parameter optimization using grid search. This approach was inferior to the RF approach, resulting in a ROC AUC of 0.52 for morbidity and 0.50 for mortality. As such, the RF outperformed the grid search optimized SVM approach, indicating adequate hyper parameter optimization for the RF approach. This information has been added to the manuscript.

Comment #10

I suggest incorporating Net Index Reclassification (NRI) for the purpose of comparing it with traditional methodologies.

Response:

Thank you for this suggestion. As suggested, we have included the NRI calculations as supplementary data, and updated the manuscript accordingly.

Comment #11
Additionally, the use of SHAP can provide a clearer understanding of the relationship between input properties and the resulting conclusion.

Response:

Thank you for this suggestion. We agree that the addition of SHAP values would add value to the manuscript. We have thus included these in the revised manuscript.

Comment#12

Reviewer #2: I commend you for taking up a comprehensive research analysis of data set and use it for prediction of post operative complications in a subset of pancreaticoduodenectomy cases. The different models formulated and tested are based on sound reasoning and parameters. I hope that, with use of similar computer-generated applications in health care research would help resolve many issues in future

Response:

Thank you for this comment!
