# Supplementary material for: Assessing the value of deep neural networks for postoperative complication prediction in pancreaticoduodenectomy patients
Source: PLoS One. 2024 Dec 30;19(12):e0316402. doi: 10.1371/journal.pone.0316402 (PMC11684602; doi:10.1371/journal.pone.0316402)
Supplement: Attachment — Submitted filename: Reponse to reviwers 2nd round.docx [file pone.0316402.s003.docx]

Copenhagen Nov 8th 2024

Dear PLOSOne editorial team

Thank you for assessing our submitted manuscript. We understand that reviews feel that questions have been addressed, but here seems to be an issue with the fact that the manuscript has previously been submitted as a preprint.

To formally adhere to the review phases, please find a point-by-point response to comments below.

Thank you again for the opportunity to resubmit our manuscript.

On behalf of the authors

Martin Sillesen MD, PhD

Assoc. Prof. of Surgery

Dep. of Organ Surgery and Transplantation
Copenhagen University Hospital, Rigshospitalet
Denmark

[Martin.Sillesen@Regionh.dk](mailto:Martin.Sillesen@Regionh.dk)

Reviewers' comments:

Reviewer's Responses to Questions

**Comment #1**

Comments to the Author

1. If the authors have adequately addressed your comments raised in a previous round of review and you feel that this manuscript is now acceptable for publication, you may indicate that here to bypass the “Comments to the Author” section, enter your conflict of interest statement in the “Confidential to Editor” section, and submit your "Accept" recommendation.

Reviewer #2: All comments have been addressed

Reviewer #3: All comments have been addressed

**Response**:

Thank you

**Comment #2**

6. Review Comments to the Author

Please use the space provided to explain your answers to the questions above. You may also include additional comments for the author, including concerns about dual publication, research ethics, or publication ethics. (Please upload your review as an attachment if it exceeds 20,000 characters)

Reviewer #2: Thank you very much for addressing the reviewers queries and concerns. I believe it would be suitable in the updated form.

Reviewer #3: This is a preprint paper from
https://www.medrxiv.org/content/10.1101/2023.08.21.23294364v1

This is a preprint paper from
<https://www.medrxiv.org/content/10.1101/2023.08.21.23294364v1>

**Response:**

It is indeed true that this manuscript has previously been submitted as a preprint, which we believe is in accordance with PLOSOne guidelines. This seems to be more of a comment pertaining to the fact this this has been presented as a preprint, rather than a request for edits from the side of the reviewer.
However, from conversations with the editorial office, we understand that there is a request from the editorial office for “…authors to make more improvements from the version in the preprint. Please revise to show more improvements.”.

The original preprint version was submitted in August 2023, whereas the present version was substantially edited based on the reviewer’s comments. As such, the preprint and the current manuscript is less than 80% identical to the preprint, indicating that 20% of the text has been updated/altered compared with the preprint.

Furthermore, based on the reviewers inputs, we have updated the manuscript with new tables and data, added new modelling approaches including benchmarking with SVMs and included parameter optimization . We have, as per reviewers suggestions, included Net Index Classification and Shapley insights into driving factors as well as performed a general editing of the manuscript text.
As such, we would respectfully argue that the current version of the manuscript is indeed very different from the preprint version.
While we are of course open to performing other changes to the manuscript, we find it difficult to make changes to a manuscript that is already significantly different from its preprint version both in terms of text, data, figures, tables and methods without more specific instructions on where the editors see the need for improvements specifically.
